# Laparoscopic resection of metachronous metastasis to the contralateral adrenal gland 5 years after radical nephrectomy

**DOI:** 10.1097/RC9.0000000000000608

**Published:** 2026-06-22

**Authors:** Kenan Mohsen Sbh, Ahmad Al-Bitar, Abdul Ghani Alshalabi

**Affiliations:** a Department of General Surgery, National University Hospital, Faculty of Medicine, Damascus University, Damascus, Syrian Arab Republic; b Faculty of Medicine, Damascus University, Damascus, Syrian Arab Republic; c Department of General Surgery, Faculty of Medicine, Damascus University, Damascus, Syrian Arab Republic

**Keywords:** clear cell renal cell carcinoma, contralateral adrenal gland, laparoscopic adrenalectomy, metachronous metastasis, renal cell carcinoma

## Abstract

**Introduction:**

Renal cell carcinoma (RCC), especially clear cell RCC (ccRCC), frequently metastasizes to the lungs and bones after nephrectomy. However, isolated metachronous contralateral adrenal metastasis is exceptionally rare (≈0.7% of cases).

**Case presentation:**

A 57-year-old male, 5 years after a left radical nephrectomy for stage I ccRCC, presented with mild right upper quadrant discomfort. Ultrasound showed a 3.5-cm right adrenal mass with no significant internal vascularity on Doppler – an atypical hypovascular finding for ccRCC metastasis. Laboratory tests confirmed a non-functional lesion. A laparoscopic right adrenalectomy was performed. Histopathology and immunohistochemistry (IHC) (PAX-8+, CA-IX+, CD10+; Melan-A–, Inhibin–, Synaptophysin–) confirmed metastatic ccRCC. Recovery was uneventful, with no recurrence observed on 2-year surveillance imaging.

**Clinical discussion:**

This case highlights the diagnostic challenge of contralateral adrenal metastases, especially when imaging is atypical for hypervascular ccRCC metastases. Definitive diagnosis requires histopathology and IHC to exclude primary adrenal neoplasms. Surgical resection is the preferred treatment for isolated metachronous metastases, offering potential cure or prolonged disease-free survival. The 5-year disease-free interval before metastasis underscores the delayed and variable recurrence patterns of RCC.

**Conclusion:**

ccRCC can present with late, solitary metastases to unusual sites like the contralateral adrenal gland years after initial treatment. A high index of suspicion and thorough pathological evaluation are essential. Minimally invasive surgical resection is effective for isolated metachronous metastases. This case report reinforces the need for long-term, vigilant follow-up of RCC survivors.

## Introduction

Renal cell carcinoma (RCC), particularly the clear cell subtype (ccRCC), constitutes approximately 90% of kidney tumors^[^1^]^, with metastases developing in about 20–40% of cases following radical nephrectomy^[^2^]^.

The most common sites for metastases are the lungs (43%) and bones (35%). Tumor infiltration into the adjacent adrenal gland or same-side metastases is relatively frequent, occurring in 3–5% of cases. However, isolated adrenal metastases from a previously removed contralateral kidney tumor are extremely rare, with an incidence of around 0.7%^[^3,4^]^.

HIGHLIGHTS

- Renal cell carcinoma, particularly the clear cell subtype, can manifest with isolated metachronous metastasis to the contralateral adrenal gland, even after a prolonged disease-free interval of 5 years or more.
- Diagnosis can be challenging due to atypical imaging features; a lack of significant vascularity on Doppler ultrasound, as seen in this case, may mimic a primary adrenal lesion and necessitate definitive confirmation via immunohistochemistry.
- Laparoscopic adrenalectomy is a viable and effective minimally invasive surgical option for managing such solitary metastases, with the potential for excellent medium-term oncologic outcomes and should be considered in suitable patients.

Previous studies have documented both synchronous and asynchronous occurrences of similar cases, often managed through surgical intervention. In this paper, we discuss a case of delayed metastasis from a renal tumor to the contralateral adrenal gland, identified after several years of clinical and radiological monitoring.

This case report has been presented in line with the SCARE checklist ^[^5^]^

## Case presentation

A 57-year-old Arab male patient presented to our department in November 2023 with a primary complaint of mild, persistent right upper quadrant abdominal discomfort of 2 months’ duration. He reported no other accompanying symptoms, such as fever, weight loss, or hematuria.

His past medical history was unremarkable. He had no known drug allergies. His family history was negative for RCC or other genitourinary malignancies. He denied any history of smoking, alcohol use, or occupational exposure to known carcinogens.

The patient’s surgical history included a radical left nephrectomy for a stage I clear cell renal cell carcinoma (ccRCC) in 2018, with tumor-free margins (3.5 cm in diameter, confined to the kidney, and no regional lymph node metastases). Adjuvant chemotherapy (six doses) was administered post-nephrectomy as part of a clinical trial protocol for localized RCC at that time. A pre-nephrectomy staging contrast-enhanced computed tomography (CT) scan of the chest, abdomen, and pelvis showed no evidence of synchronous metastases or bilateral adrenal abnormalities. The patient was followed up with clinical examination, serum creatinine monitoring, and annual abdominal ultrasound for 5 years, with no signs suggestive of local recurrence or metastatic disease. A detailed timeline is provided in Table 1.

**Table 1:** Clinical timeline.

Date/period	Event
2018	Left radical nephrectomy for stage I ccRCC + adjuvant chemotherapy
2018–2023	Regular surveillance; no evidence of disease
September 2023	Onset of mild right upper quadrant discomfort
November 2023	Presentation to our department; imaging revealed right adrenal mass
November 2023	Laparoscopic right adrenalectomy
November 2023–present (2 years follow-up)	No recurrence on surveillance imaging

Upon hospital admission, clinical examination showed mild tenderness in the right flank but no palpable masses, organomegaly, or lymphadenopathy. Vital signs were normal.

## Diagnostic assessment

**Figure 1:**
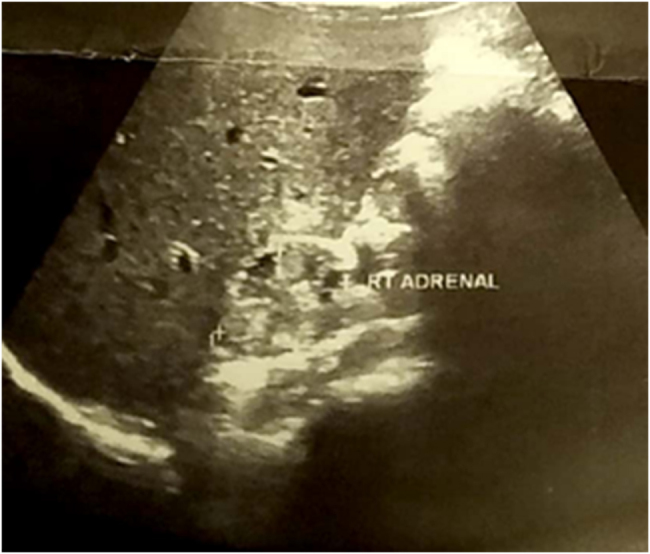
Abdominal ultrasound shows a hyperechoic, heterogeneous, 3.5-cm right adrenal mass.

An abdominal ultrasound revealed a 3.5-cm hyperechoic and heterogeneous mass in the right adrenal gland. Color Doppler imaging showed no significant internal vascularity (Fig. 1), a finding not typical of the hypervascularity often seen in ccRCC metastases.

**Figure 2.**
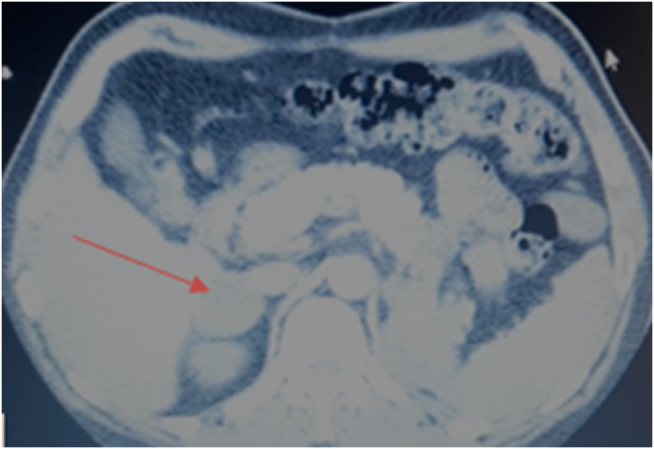
Contrast-enhanced CT scan of the abdomen showing an enhanced right adrenal mass (arrow).

A contrast-enhanced abdominal and pelvic CT scan was then performed, revealing a well-defined mass in the right adrenal gland measuring 38 × 42 × 33 mm with central necrosis (Fig. 2). No other metastatic lesions were identified in the liver, lungs, or retroperitoneum.

Laboratory examination confirmed that the adrenal mass was non-functional. Serum cortisol (8 AM: 12 μg/dL, normal 5–25), aldosterone (5 ng/dL, normal 2–9), plasma metanephrines (0.3 nmol/L, normal <0.5), and renin (1.0 ng/mL/h, normal 0.5–2.0) were within normal limits. Renal function was normal (creatinine: 1.0 mg/dL; eGFR > 90 mL/min).

## Operative management

The patient was prepared for surgery and underwent a laparoscopic right adrenalectomy under general anesthesia using a lateral transabdominal approach with three ports. Intraoperatively, the right adrenal gland contained a well-circumscribed, firm, yellowish mass without invasion into the liver, inferior vena cava, or surrounding retroperitoneal structures. The adrenal vein was ligated with Hem-o-Lok clips, and the specimen was removed in an endoscopic retrieval bag. Estimated blood loss was less than 50 mL, and operative time was 90 minutes. No intraoperative complications occurred.

## Postoperative course

The patient was hospitalized for 24 hours for pain control and monitoring. He tolerated oral intake and ambulated on the evening of surgery. He was discharged in good general condition on postoperative day 1 with no complications.

## Histopathology and immunohistochemistry

**Figure 3.**
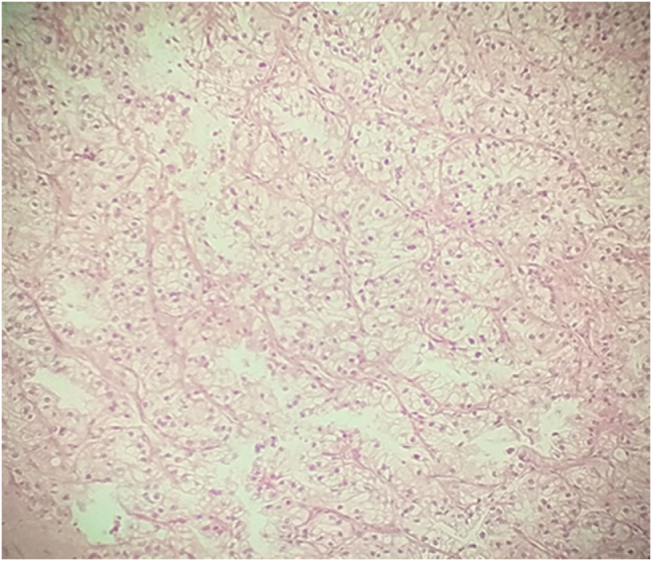
Histological sections of the adrenal mass showing clear cell type of renal cell carcinoma (RCC) adrenal metastasis.

The resected specimen was a well-circumscribed, ovoid mass measuring 4.0 × 3.5 × 3.0 cm. On sectioning, it exhibited a variegated cut surface with areas of yellow-tan coloration and central necrosis. Histopathological examination confirmed the presence of adrenal tissue infiltrated by metastatic grade 2 ccRCC. Immunohistochemistry was strongly positive for PAX-8, CA-IX, and CD10, and negative for Melan-A, Inhibin, and Synaptophysin, confirming the diagnosis of metastatic ccRCC and effectively excluding adrenocortical carcinoma, pheochromocytoma, and other primary adrenal tumors (Fig. 3).

The patient recovered well postoperatively. Surveillance imaging (contrast-enhanced CT scans of the chest, abdomen, and pelvis) at 6-month intervals for 2 years has shown no evidence of local recurrence or new metastatic disease. The patient remains asymptomatic and has returned to full activity.

## Discussion

RCC, particularly the clear cell subtype (ccRCC), accounts for approximately 90% of kidney tumors^[^1^]^ and is characterized by a high propensity for metastasis, most commonly affecting the lungs, lymph nodes, bones, liver, brain, and adrenal glands^[^6^]^. Isolated contralateral adrenal metastases, however, remain a rare clinical occurrence^[^7^]^.

The present case is notable for three reasons: (1) an exceptionally long disease-free interval of 5 years following resection of a stage I primary tumor; (2) atypical hypovascular imaging features on Doppler ultrasound, which initially suggested a primary adrenal lesion rather than a ccRCC metastasis; and (3) successful laparoscopic management with 2 years of disease-free survival.

Renal metastases can occur either synchronously or metachronously. Studies have reported late and solitary metastases many years after nephrectomy [8,9]. In the present case, metastasis was detected 5 years after curative surgery for a stage I primary tumor. The primary tumor in 2018 was a stage I clear cell RCC. The adjuvant chemotherapy administered post-nephrectomy was part of a clinical trial protocol, reflecting the evolving therapeutic landscape at the time, though it is not the current standard of care for localized RCC. Delayed metastases are attributed to factors including a low mitotic index and an impaired immune response, which can allow tumor cells to persist and spread undetected^[^8,9^]^. Recent evidence suggests that dormant tumor cells may reside in the bone marrow or other niches for years before reactivation, a phenomenon increasingly recognized in ccRCC^[^10^]^. The spread of tumor cells follows primary pathways: local infiltration, hematogenous spread, and lymphatic dissemination^[^11,12^]^. Nevertheless, the precise biological pathway predisposing to isolated secondary involvement of the contralateral adrenal gland by RCC remains unclear.

The diagnosis of contralateral adrenal metastasis poses a significant radiological and pathological challenge, primarily due to the need to differentiate it from more common primary adrenal neoplasms, such as adenoma or adrenocortical carcinoma. Radiologically, ccRCC metastases are typically hypervascular. In contrast, the mass in our case demonstrated no significant internal vascularity on Doppler ultrasound, which is an atypical presentation and more suggestive of a hypovascular primary adrenal lesion. This case illustrates that imaging characteristics can be variable and sometimes misleading; reliance on classic hypervascular features alone would have delayed diagnosis. Studies highlight that renal metastases are distinguished by their vascular nature, in contrast to adrenal adenomas and adrenal carcinomas, which tend to be hypovascular. This difference in vascularization has a significant impact on imaging characteristics, diagnostic accuracy, and potential therapeutic approaches^[^13^]^. Therefore, when a hypovascular adrenal mass is encountered in a patient with a history of RCC, metastatic disease should remain in the differential diagnosis, and tissue confirmation is strongly recommended. Definitive diagnosis often hinges on histopathological examination supplemented by immunohistochemistry (IHC). In our case, IHC profiling (positive for PAX-8, CA-IX, and CD10; negative for adrenal cortical markers like Melan-A and Inhibin) was crucial to confirm the metastatic renal origin and rule out a primary adrenal malignancy. The use of PAX-8 is particularly valuable as it is a sensitive and specific marker of renal tubular origin, even in poorly differentiated metastases^[^14^]^.

Surgical resection remains the preferred and potentially curative treatment for isolated metachronous metastases^[^6^]^. Several cases of late metastases to the contralateral adrenal gland have been published^[^13–15^]^, with the longest duration of follow-up being 23 years after nephrectomy^[^16^]^. The laparoscopic approach offers advantages, including shorter hospital stay, reduced blood loss, and faster recovery, provided that the mass is localized and non-invasive^[^17^]^. Our patient underwent successful laparoscopic resection and has remained disease-free for 2 years post-adrenalectomy, supporting the role of metastasectomy in selected cases. In the era of targeted therapies and immune checkpoint inhibitors for metastatic RCC, surgical metastasectomy remains an important component of oligometastatic disease management, potentially offering prolonged disease-free survival or even cure^[^18^]^.

## Limitations

This case report has inherent limitations, including its single-patient nature and the lack of high-resolution preoperative CT imaging for publication (Fig. 2 due to the conflict in Syria). Additionally, longer follow-up is needed to confirm durable remission, as late recurrences beyond 2 years have been well documented in RCC.

## Conclusion

This case report contributes to the literature by illustrating several key clinical points: (1) the exceptionally long (5-year) disease-free interval prior to solitary metastasis, highlighting the unpredictable biology of RCC; (2) the diagnostic challenge posed when imaging features are atypical for ccRCC metastasis, necessitating a high index of suspicion and definitive pathological confirmation with IHC; and (3) the excellent medium-term oncologic outcome achievable with minimally invasive surgical resection of such isolated late recurrences.

## Data Availability

Not applicable.
